# Surgical Principles for Children/Adolescents With Newly Diagnosed Rhabdomyosarcoma: A Report from the Soft Tissue Sarcoma Committee of the Children's Oncology Group

**DOI:** 10.1080/1357714021000066359

**Published:** 2002-12

**Authors:** David A. Rodeberg, Charles N. Paidas, Thom L. Lobe, Kenneth Brown, Richard J. Andrassy, William M. Crist, Eugene S. Wiener

**Affiliations:** Division of Pediatric Surgery Mayo Clinic 200 First Street SW Rochester MN 55905 USA


*Sarcoma* (2002) **6,** 111–122

Taylor & Francis healthsciences

**REVIEW**

### Surgical principles for children/adolescents with newly diagnosed rhabdomyosarcoma: a report from the Soft Tissue Sarcoma Committee of the Children's Oncology Group

DAVID A. RODEBERG, CHARLES N. PAIDAS, THOM L. LOBE, KENNETH BROWN, RICHARD J. ANDRASSY, WILLIAM M. CRIST, & EUGENE S. WIENER

*Division of Pediatric Surgery, Mayo Clinic, 200 First Street SW, Rochester, MN 55905, USA*

### Introduction

Rhabdomyosarcoma (RMS) is one of the more common solid tumors in children, with approximately 250 new cases diagnosed each year.[1] The roles of pediatric surgeons in the treatment of RMS have changed significantly through the years, as other adjuvant therapies have become more efficacious. The purpose of this manuscript is to describe the current surgical therapy recommendations of the Soft Tissue Sarcoma Committee of the Children's Oncology Group (COG), formerly known as the Intergroup Rhabdomyosarcoma Group (IRSG).

### Background

The first described case of RMS was by Webner in 1854,[2] but the first report of RMS in children was not until 1952, by Pack *et al.*[3] During those early years, surgery was the only therapy used, and often involved radical excision of tumor and normal tissue, including amputation and exenteration. Even with that aggressive surgical intervention, survival rates of only 7–70%, depending on tumor site, were achieved.[4,2] In 1961, the addition of high-dose chemotherapy resulted in a significant improvement in survival. In 1965, the addition of high-dose radiation therapy with chemotherapy and surgical excision also led to an improvement in patient survival. Noting that chemotherapy, radiation, and surgery all had a part in the treatment of RMS, the Intergroup Rhabdomyosarcoma Study Group (IRSG) was formed in 1972. This group was charged with developing a multidisciplinary approach to the treatment of RMS.[7] Since its inception, IRSG has enrolled over 3000 patients and has significantly improved overall survival rates for RMS. Currently, IRSG is enrolling patients in IRS-V.

Surgeons continue to have a pivotal role in the treatment of RMS. The goal of this manuscript is to provide guidelines to surgeons for the care of RMS patients. Surgeons are involved in the preoperative staging and postoperative grouping of patients, as well as biopsy and excision of tumors.

### Epidemiology

Rhabdomyosarcoma is not an uncommon tumor, accounting for 5% of all pediatric solid tumors.[2] There are approximately 4 million cases/million population/year;[1] 250 new cases of RMS in the United States each year. The median age at presentation is 5 years; however, this seems to follow a bimodal distribution with peak incidences between 2 and 4 years and between 12 and 16 years. Therefore, approximately 80% of RMS cases have occurred by 14 years of age.[8] There is a slight male preponderance, with a male to female ratio of 3 to 2.

From the Intergroup Rhabdomyosarcoma Study Group (IRSG) representing the Children's Cancer Group[1], the Pediatric Oncology Group[2], and the Intergroup Rhabdomyosarcoma Statistical Office[3] including; James R. Anderson, PhD, Richard J. Andrassy, MD, Carola A.S. Arndt, MD, K. Scott Baker, MD, Frederic G. Barr, MD, W. Archie Bleyer, MD, Philip Breitfeld, MD, John C. Breneman, MD, Julia Bridge, MD, Kenneth Brown, MD, William M. Crist, MD, Sarah S. Donaldson, MD, Holcombe E. Grier, MD, Douglas Hawkins, MD, Peter J. Houghton, PhD, Michael Link, MD, Thom L. Lobe, MD, Harold M. Maurer, MD, William H. Meyer, MD, Jeff Michalski, MD, Sharon Murphy, MD, Charles N. Paidas, MD, Alberto S. Pappo, MD, David M. Parham, MD, Stephen J. Qualman, MD, R. Beverly Raney, MD, Leslie Robison, PhD, Eric Sandler, MD, Stephen Skapek, MD, Lynn Smith, MD, Poul H.B. Sorensen, MD, PhD, Sheri Spunt, MD, Lisa Teot, MD, Timothy Triche, MD, PhD, Teresa J. Vietti, MD, David Walterhouse, MD, Moody Wharam, MD, Eugene S. Wiener, MD, Suzanne Wolden, MD, Richard Womer, MD.

This work was supported in part by NIH/NCI Grants: CA24507 and CA72989.

Correspondence to: David A. Rodeberg, M.D., Division of Pediatric Surgery, Mayo Clinic, 200 First Street SW, Rochester, MN 55905, USA. Tel.: +1-507-284-8391; Fax: +1-507-284-0058; E-mail: rodeberg.david@mayo.edu

ISSN 1357–714X print/ISSN 1369–1643 online/02/0200111–12 © Taylor & Francis Ltd
DOI: 10.1080/1357714021000066359

The exact etiology of RMS is unknown, but there appears to be some genetic predisposition. Certain risk factors have been identified, including Li Fraumeni syndrome, in which patients present with RMS at an early age and have a family history of other carcinomas, especially premenopausal breast carcinoma.[9] This syndrome is an autosomal-dominant disorder and is usually associated with a germline mutation of p53.[10] Other possible risk factors for RMS include neurofibromatosis, nevoid basal cell carcinoma, fetal alcohol syndrome, and maternal exposure to marijuana or cocaine, X-rays, and employment as a healthcare worker.[11–14]

### Pathology

Rhabdomyosarcomas arise from primitive mesenchymal cells that are present throughout the body, even in areas that are usually not associated with striated muscle.[15] However, all tumors show some degree of striated muscle differentiation. Rhabdomyosarcomas may invade local structures and frequently metastasize early through lymphatics or hematogenous spread. Tumors are usually firm, nodular, and variable in size and consistency; however, they are not encapsulated and invade surrounding soft-tissue structures. There are essentially four types of RMS: embryonal, alveolar, pleomorphic, and undifferentiated.[7] Embryonal is the most common and is usually found in children less than 8 years old. Embryonal also constitutes 80% of all genitourinary (GU) RMS and 60% of head and neck RMS. Within the embryonal type, there are two subvariants including spindle-cell RMS, which is common in paratesticular lesions, and botryoid RMS, which is found in mucosa-lined hollow viscera. Overall, patients with embryonal RMS have good prognoses, with a 5-year survival rate of 60%. For spindle cell and botryoid subvariants the survival rate increases to 95%. Alveolar RMS is found in older children and is associated with tumors in the extremities and trunk. Patients with alveolar RMS have slightly worse prognoses than those with embryonal, with an average 5-year survival rate of 54%. Pleomorphic RMS also involves the limbs and trunk; however, patients with it have better prognoses than those with alveolar, with a 75% 5-year survival rate. Patients with undifferentiated RMS have the worst prognoses, with a 40% survival rate and tumors in the limbs, head, and neck areas.

### Preoperative evaluation

### *Presentation*

Most RMS tumors present as asymptomatic masses detected by patients or their families. Some tumors present with signs and symptoms that vary according to primary tumor origin and may be secondary to mass effect or complications of the tumor.

### *Preoperative workup*

Patients with suspected RMS require a complete workup before definitive surgery. This includes standard blood work such as complete blood counts (CBC), electrolytes and renal function tests, liver function tests (LFTs), and urinalysis (UA). There are no serum tumor markers for RMS. The primary tumor should be evaluated with computer tomography (CT) or magnetic resonance imaging (MRI).[16] MRI provides better definition of the tumor and surrounding structures, therefore is preferable, especially for limb, pelvic, and paraspinal lesions. CT is probably better for evaluation of bone erosion and abdominal adenopathy.[17] Metastatic evaluation includes a bone marrow aspirate and bone scan, CT of the brain, lungs, and liver, and lumbar puncture for cerebrospinal fluid (CSF) collection. Metastatic tumor can also be detected using a gallium scan.[18]

### *Pretreatment clinical staging*

This is a modification of the TNM staging system and is based on primary tumor site, primary tumor size, clinical regional nodal status, and distant spread[19,20] (Table 1). These criteria were the best predictors of survival in nonmetastatic patients during the IRSG II study. Staging is based on clinical findings, such as preoperative imaging and physical findings, and should be done by surgeons and oncologists. Size of the tumor should be determined by physical examination or imaging measurements. Careful evaluation of clinical and imaging findings is imperative before assignment of primary tumor site because site designation alters stage and treatment assignment. Intraoperative findings and pathological results should not affect stage (but will affect Clinical Group).

### Surgical principles

### *Biopsy*

Frequently the initial procedure for patients with a mass suspected to be RMS is a biopsy, usually open, which obtains an adequate specimen for pathological, biological and treatment protocol studies. There may be instances when core needle biopsy is appropriate, such as metastatic disease or small lesions in areas that will be treated primarily by chemotherapy and radiotherapy.[21,22]

### *Neoadjuvant therapy*

After staging studies some tumors will be declared unresectable because of their large size or approximation to vital structures. Neoadjuvant therapy may shrink tumors, converting unresectable tumors to resectable or decreasing resection morbidity. Results of IRSG studies have shown the efficacy of chemotherapy for tumor shrinkage and subsequent

**Table 1.** *TNM pretreatment staging classification. Staging before treatment requires thorough clinical, laboratory, and imaging examinations. Biopsy is required to establish histological diagnosis. Pretreatment tumor size is determined by external measurement or MRI or CT, depending on anatomic location. For less accessible primary sites, CT also will be used for lymph node assessment. Metastatic sites will require some form of imaging (but not histological confirmation, except for bone marrow examination) confirmation.*

| Stage | Sites | T | Size | N | M |
|---|---|---|---|---|---|
| 1 | Orbit<br>Head and neck (excluding Parameningeal)<br>GU nonbladder/ Nonprostate | $T_1$ or $T_2$ | a or b | $N_0$ or $N_1$ or $N_x$ | $M_0$ |
| 2 | Bladder/Prostate, Extremity, Cranial Parameningeal, Other (Includes trunk, Retroperitoneum, etc.) | $T_1$ or $T_2$ | a | $N_0$ or $N_x$ | $M_0$ |
| 3 | Bladder/Prostate, Extremity, Cranial Parameningeal, Other (Includes trunk, Retroperitoneum, etc.) | $T_1$ or $T_2$ | a<br>b | $N_1$<br>$N_0$ or $N_1$ or $N_x$ | $M_0$<br>$M_0$ |
| 4 | All | $T_1$ or $T_2$ | a or b | $N_0$ or $N_1$ | $M_1$ |

| | | | |
|---|---|---|---|
| Definitions | *Tumor* | $T(site)_1$ | confirmed to anatomic site of origin<br>(a) <5 cm in diameter; (b) >5 cm in diameter |
| | | $T(site)_2$ | Extension and/or fixative to surrounding tissue<br>(a) <5 cm in diameter; (b) >5 cm in diameter |
| | *Regional nodes* | | |
| | | $N_0$ | regional nodes not clinically involved |
| | | $N_1$ | regional nodes clinically involved by neoplasm |
| | | $N_x$ | clinical status of regional nodes unknown (especially sites that preclude lymph node evaluation) |
| | *Metastasis* | | |
| | | $M_0$ | no distant metastasis |
| | | $M_1$ | metastasis present |

resection.[23,24] In these studies, Group III patients treated with chemotherapy followed by complete excision had prognoses similar to Group I patients.

### *Node sampling or dissection*

Clinical or radiographic evaluation of regional lymph nodes should be done during diagnostic workup and is an important component of pretreatment staging (Table 2). Clinically positive nodes should always be confirmed pathologically. Open biopsy is recommended; however, core needle biopsy or fine needle aspiration may be appropriate, based on the surgeon's judgment and pathologist's recommendations.[25,26] For multiple clinically positive nodes, radical debulking may be useful, with radiotherapy, to obtain regional control.[27,28] During biopsy of the regional lymph nodes, a 'distant' node should be harvested for pathological study. For upper extremity lesions this would consist of an ipsilateral supraclavicular (scalene) biopsy, for the lower extremity an iliac and/or para-aortic node biopsy, and for paratesticular the ipsilateral para-aortic lymph node at the renal vein. Involvement of these distant nodes is analogous to metastatic disease.[27] There is no benefit from formal nodal dissection if distant node or metastatic disease has already occurred.

Pathological evaluation of clinically uninvolved nodes is site specific; it is required in extremity sites[27] and for children older than 10 years with paratesticular tumors (manuscript in preparation). Aggressive regional lymph node sampling is the most appropriate method of surgical evaluation since resection is diagnostic but not therapeutic. For this reason, prophylactic radical node dissection, as used for some other malignancies, is not necessary in childhood rhabdomyosarcoma.

Sentinel node mapping using blue dye, radioactive-labeled colloid or both is helpful in determining regional node status.[29] Preliminary data from the IRSG suggest that sentinel node biopsy may be effective (unpublished). We anticipate that sentinel lymph node biopsy will become the standard of care for the next IRSG study.

### *Margins*

The basic principles of wide and complete resection of the primary tumor with a surrounding 'envelope' of normal tissue should be followed at the initial, or subsequent, operations whenever possible. A somewhat arbitrary margin of 0.5 cm circumferentially, or an uninvolved fascia margin, are adequate. This size of margin generally is more easily obtained in the

**Table 2.** *Regional nodal basins for rhabdomyosarcoma*

| |
|---|
| *Extremity* |
| Lower extremity-inguinal, femoral, popliteal nodes (rarely involved) |
| Upper extremity-axillary, brachial, epitrochlear, infraclavicular nodes (infraclavicular) |
| *Genitourinary* |
| Bladder/prostate-pelvic, retroperitoneal nodes at renal artery level or below |
| Cervix and uterus-pelvic, retroperitoneal nodes at renal artery level or below |
| Paratesticular-pelvic, retroperitoneal nodes at renal artery level or below |
| Vagina-retroperitoneal, pelvic nodes at or below common iliacs inguinal nodes |
| Vulva-inguinal nodes |
| *Head and neck* |
| Head/neck-ipsilateral cervical, jugular, preauricular, occipital, supraclavicular nodes for laterally placed tumors (excluding scalp); may have bilateral adenopathy with centrally placed tumors |
| Orbit/eyelid-ipsilateral jugular, preauricular, cervical nodes |
| *Intrathoracic* |
| Intrathoracic-internal mammary, mediastinal nodes |
| *Retroperitoneum/pelvis* |
| Retroperitoneum/pelvis-pelvic, retroperitoneal nodes |
| *Trunk* |
| Abdominal wall-inguinal, femoral nodes |
| Chest wall-axillary, internal mammary, infraclavicular nodes |
| *Other* |
| Biliary-liver hilar nodes |
| Perianal/perineal-inguinal, pelvic nodes; may be bilateral |

*Notes:* any tumor-involved node other than those listed above signifies distant metastasis (Stage 4/Group IV). Examples: perineal primary with nodes above the pelvis; thigh primary with iliac or periaortic nodes; intrathoracic primary with subdiaphragmatic nodes; paratestis primary with inguinal nodes with or without trans-scrotal biopsy or scrotal involvement. We would like to thank Dr. Bev Raney for use of this table.

extremities or trunk than in head and neck tumors. Adequate margins of uninvolved tissue are required unless excision involves sacrifice of normal tissue that cannot be resected, would result in an unacceptable loss of function/cosmesis, or is not technically feasible. The surgeon should mark all margins and orient the specimen at the operative field, so that margin evaluation is precise. Narrow margins are unavoidable in some sites. In those situations, the surgeon should take several separate biopsies of the 'normal' tissue around the margins of resection and these should be marked and submitted separately for pathological review. Communication with the pathologist is mandatory to assure accuracy of margin examination. The tumor should not be bisected or cut into separate specimens before being sent to the pathologist. Any suspected microscopic or gross residual tumor should be marked in the tumor bed with small titanium clips to aid radiotherapy simulation and subsequent re-excision. Adequate margins of normal tissue are preferable to leaving gross or microscopic residual diseases in all circumstances. A clear margin and no residual disease (Group I) is superior to microscopic margins (Group II) or gross residual disease (Group III) in all outcomes analyses.[30,31,24]. Exceptions to this operative approach would be primaries in the orbit, head and neck, biliary, and GU sites.[32–36] Also, when the RMS arises from a somatic muscle, excision of the entire muscle of origin or the entire compartment usually is not necessary.[37]

The initial surgical procedure may have been done before the diagnosis of RMS was established. This frequently results in an incisional biopsy or a limited excision of the RMS mass similar to that used for benign tumors. This situation frequently results in gross residual tumor, microscopically involved margins, or uncertainty about the margins. Under these circumstances, pretreatment re-excision (PRE) is advisable. PRE is a wide re-excision of the previous operative site, including an adequate margin of normal tissue, with careful examination of all margins before adjuvant therapy. PRE is particularly applicable to extremity and trunk lesions, but should be applied whenever feasible (unless re-excision involves sacrifice of normal tissue that cannot be resected, would result in an unacceptable loss of function/cosmesis, or is not technically feasible). PRE improves failure-free survival and, more importantly, overall survival.[30]

### Clinical group

Clinical group assignment is determined postoperatively based on pathology examination from the definitive operation. Grouping results in the stratification of patients based on completeness of excision

**Table 3.** *Intergroup rhabdomyosarcoma study clinical grouping system*

| | |
|---|---|
| Group I | Localized disease, completely resected |
| | A. Confirmed to organ or muscle of origin |
| | B. Infiltration outside organ or muscle of origin; regional nodes not involved |
| Group II | Compromised or regional resection including |
| | A. Grossly resected tumors with microscopic residual tumor |
| | B. Regional disease, completely resected, with nodes involved and/or tumor extension into an adjacent organ |
| | C. Regional disease, with involved nodes, grossly resected, but with evidence of microscopic residual tumor |
| Group III | Incomplete resection or biopsy with gross residual disease remaining |
| Group IV | Distant metastases present at onset |

and lymph node status (Table 3). Stratification of clinical groups correlates closely with long-term survival and prognosis.[38] Patients with an incisional biopsy are considered Group III if no further surgery is performed. However, for patients who undergo PRE, the final Group is based on the pathological results from the PRE.

### Secondary procedures

Operating again to determine response status after chemotherapy and radiotherapy may be considered for patients who are deemed to have partial responses, and selected nonresponders. The purpose of second-look operations is to confirm clinical response, to evaluate pathological response, and to remove residual tumor to achieve local control.[39,40] CT or MRI should be used to evaluate patients for residual local and metastatic disease. In IRS III, 75% of patients classified as having partial responses by imaging were found to have complete responses during second look operations or were converted to complete responses by excision of residual tumors.[41] Converting them to complete responses improved their survival. The second look operations were most effective in extremities and trunks compared with head and neck lesions. Flaps and/or grafts may be required for reconstruction, especially because prior radiation can affect wound closure and healing. For intracavitary sites, such as abdomen or thorax, a complete second-look evaluation using open, laparoscopic, or thorascopic evaluation can be done. If imaging or clinical evidence of residual disease exists but total excision is not possible, diagnosis should be confirmed by biopsy. However, complete resection should be the goal of the second-look operation.

### Chemotherapy

Chemotherapy was recognized as an important adjunct to surgery in the 1980s[42,43] and today all patients with RMS receive some form of adjunctive chemotherapy. The standard is a combination of vincristine, actinomycin, and cyclophosphamide (VAC). Some patients with more favorable disease receive only VA.

### Radiotherapy

Radiotherapy is an important adjunct for local control of RMS. Currently all patients receive 40–60 Gy unless they have group I nonalveolar RMS or tumors of the GU tract (vagina, uterus, vulva) that are excised completely after chemotherapy. The XRT port includes the tumor bed plus a 2-cm margin and should include the lymph node basin if nodal metastatic disease has been found.

### Specific anatomic sites

There are some site-based pathological and biological variations between tumors that require differences in surgical management.

### *Head and neck (superficial nonparameningeal)*

Head and neck lesions encompass orbital, parotid, buccal, laryngeal, and oropharyngeal locations. Wide excision is appropriate when feasible, but the possibility of achieving wide margins is restricted generally to patients with relatively superficial lesions who present early. Cosmetic and functional factors always should be considered. For some tumors, such as parotid, laryngeal, oropharyngeal and other deep tumors, surgery is limited to biopsy followed by chemotherapy and XRT for tumor eradication.[44] This treatment scheme results in good survival rates of 83%.[45,46] However, for most tumors the standard combination of surgery, chemotherapy, and radiotherapy are applicable, with the same surgical principles of complete excision used for other sites.[47–49] The incidence of cervical node involvement is quite low in head and neck primaries.[28] Cervical lymph node biopsy is not required unless nodes are involved clinically. RMS of the orbit is, in many respects, quite different from that arising in other head and neck sites. The prognosis is better and biopsy followed by chemotherapy and radiotherapy has become the standard of care.[50–52]

### *Parameningeal*

Similar to other head and neck lesions, wide excision is appropriate when feasible, but achieving wide margins is usually only possible for patients with relatively superficial lesions. Craniofacial resection for anterior skull-base tumors of the nasal areas, paranasal sinuses, temporal fossa, and other such sites should be reserved for expert surgical teams.[48] Resection also should be limited to secondary procedures when tumors persist after initial chemotherapy and radiotherapy. Cervical lymph node biopsy is not required unless nodes are involved clinically.

### *Trunk*

The category 'trunk' RMS includes paraspinal, thoracic, intra-abdominal, retroperitoneal and abdominal wall locations. These lesions frequently have alveolar histologies (40% alveolar, 20% embryonal) and like extremity lesions poor responses to chemotherapy with subsequent poor prognoses, with an over-all 5-year survival rate of 50%.[53] Unlike extremity lesions, truncal RMS has a lower incidence of nodal metastatic disease and is more likely to have local recurrence.

### *Paraspinal*

Paraspinal RMS excision is frequently incomplete because of tumor proximity to the vertebral column and spinal canal, and large tumor size at presentation (usually greater than 5 cm).[54] Patients with large, bulky, unresectable tumors may benefit from neoadjuvant chemotherapy followed by resection.[55] Regardless of neoadjuvant therapy, if patients have postoperative micro- or macroscopic residual disease, they will require XRT. However, if a paraspinal tumor does respond to induction chemotherapy, then a second-look operation with wide local excision should be done to completely excise the tumor and obviate the need for XRT.

### *Abdominal wall*

Most abdominal wall primaries can be removed completely, either at presentation or during a second-look operation after neoadjuvant chemotherapy. Excision should include the full thickness of the abdominal wall, including peritoneum and overlying skin, *en bloc* resection of any local extension, and a margin of normal tissue. A recent study by Beech *et al.* suggested that long-term outcomes for these patients is affected by micro- or macroscopic residual disease, age, and alveolar histology. If size or location of tumors does not allow adequate excision, then initial biopsy should be followed by chemotherapy with subsequent evaluation for excision after 3 months of therapy. If complete resection is accomplished at diagnosis or second-look operation, no postoperative XRT is required. Abdominal wall reconstruction can be done using mesh or myocutaneous muscle flaps, with excellent results. These repairs can generally be accomplished while preserving good function and cosmesis.

### *Chest wall*

Many other types of sarcomas, such as Ewing or primitive neuroectodermal tumor (PNET), can present as chest-wall masses. Therefore, initial biopsies should be done for all chest-wall tumors. The proper subsequent therapy is determined by histology. The biopsy should always be done on the long axis of the tumor, which is parallel to the ribs. Once a diagnosis of RMS is established, unless complete excision is believed to be feasible, chemotherapy should be initiated. After 3 months of chemotherapy, patients can be evaluated with imaging studies to consider complete tumor excision. Thoracoscopy is sometimes beneficial to determine pleural extent of tumors and the presence of attachments to underlying lung. An excision should be wide, removing the full thickness of the chest wall, including the previous biopsy site, involved chest wall muscles, involved ribs, and may require wedge excision of underlying lung. It is not necessary to remove the entire length of the rib. Also, it is not necessary to remove the rib above and below if a wide margin can otherwise be accomplished. Sometimes removal of the periosteum of the rib above and below will allow adequate margins while preserving the rib. Because of high local recurrence rates it has been recommended that wider margins than usual should be attempted, preferably 2 cm. Reconstruction can be accomplished with mesh or myocutaneous muscle flaps. Sometimes bone struts are necessary, using homografts, rib from the contralateral side, or titanium rib implants. If the tumor is completely removed with no macro or microscopic residual disease, no postoperative radiotherapy is required. However, there has been some suggestion, given the high recurrence rate of chest-wall disease, that adjuvant XRT may be beneficial for all patients.[57] Chest-wall lesions have worse prognoses than other trunk lesions because of high local and distant recurrence rates, with a 1.8-year survival rate of only 42%.[57]

### *Retroperitoneum/pelvis*

RMS of the retroperitoneum and pelvis is usually large and the exact site of origin is difficult to determine. These tumors usually are so large and envelop so many vital structures they are unresectable at presentation; however, the same basic surgical principles apply. Patients with tumors believed resectable should undergo complete excision. When successful initial resection is unlikely, biopsy for diagnosis should be performed followed by chemotherapy and secondary resection attempts. Success with aggressive resection, including vena cava and aorta, has been reported.[58] Aggressive resection should not be done for patients with Group IV metastatic disease because survival is not improved in these patients by primary tumor resection. However, group IV patients with embryonal histologies, particularly those under 10 years old, do relatively well regardless of residual disease. This conclusion stems from the observation that there is improved failure-free survival if surgical debulking is done during initial surgery, rather than biopsy alone.[59] Therefore, in this site, for this patient age, and with embryonal histologies only, tumor debulking without sacrifice of important structures or function can be considered.

### *Biliary*

Patients with tumors of the biliary system do relatively well with chemotherapy and radiotherapy and without aggressive surgery because they usually have the botryoid variant of embryonal RMS histology.[35,60,61] The primary role of surgery often is for diagnosis and staging. If tumor excision can be accomplished without radical resection it should be attempted because survival is improved for patients with microscopic residual disease compared to macroscopic or gross residual disease. Relief of biliary obstructions is likely with chemotherapy; therefore attempts to establish biliary drainage with aggressive intervention are unnecessary. Likewise, external drainage of the biliary system while the patient is immunosuppressed with chemotherapy is associated with a high incidence of sepsis.[35]

### *Perineum/perianal*

Perineal and perianal RMS are often large tumors (greater than 5 cm). There is 100% failure-free survival with completely resected Group I tumors compared with 24% for metastatic disease group IV tumors (Blakely ML, submitted). Thus, if complete resection while maintaining function is feasible, it should be attempted. Long-term patient survival declines dramatically as the amount of residual disease and patient age increases. The same surgical principles for other trunk sites apply to these lesions. Occasionally, a temporary colostomy is necessary if there is anorectal obstruction.

### *Extremity*

Extremity RMS is characterized by a higher incidence (50%) of patients with alveolar histology. Alveolar RMS is most common in the proximal lower extremities. Regional lymph nodes are positive in 10–25% of patients. This high rate of nodal metastatic disease negatively affects survival, with a 46% survival rate for node-positive patients compared with 80% for node-negative.[62,63]

Extremity tumors often can be widely resected while sparing the involved limb. Radical soft tissue or compartmental excision generally provides a wide margin that is sufficient for local tumor control. Excision of an entire muscle from origin to insertion or resection of an entire compartment may not be required, depending on the size and invasiveness of the specific tumor. Rarely is amputation required for tumor excision. The importance of complete excision cannot be overstated because survival is 70–91% with microscopic residual disease or complete excision and only 23–50% with metastatic or gross residual disease.[62,67] Also, the survival rate for incomplete resection is no better than incisional biopsy alone.[57] Approximately half of children who die from these tumors have local recurrences;[64] therefore, if residual tumor is known or suspected after initial resection, a PRE should be done to excise the tumor before other treatment is begun.

Regional lymph node evaluation is integral in staging patients with extremity RMS.[27] Systematic, aggressive inguinal or axillary lymph node sampling is required even when there are no clinically involved nodes. Axillary dissection with preservation of pectoral muscles, long thoracic nerve, and thoracodorsal nerve has low morbidity and is the best sampling procedure for upper extremity lesions. Femoral triangle node sampling, rather than a formal node dissection, is recommended for lower extremity lesions. If nodes are involved clinically, biopsy of more central nodes should be done before regional sampling. For lesions of the upper extremity this involves ipsilateral supraclavicular (scalene) node biopsy and for the lower extremity, iliac or para-aortic node biopsy. Involvement of these central nodes is considered distant metastatic disease (Clinical Group IV) rather than regional involvement, and therefore will be important in determining subsequent adjuvant therapy and prognosis. If regional nodes are involved then XRT fields are adjusted to incorporate regional lymph nodes. Incorporation of disease-positive regional nodes into the XRT field is associated with lower local and regional recurrence rates.[65]

### *Genitourinary: bladder/prostate*

RMS of the bladder and prostate usually is large, unresectable, and of embryonal histology. Bladder salvage is an important goal of therapy and can be anticipated in 50–60% of patients.[26] Tumors rarely may be completely resectable at presentation, with preservation of bladder and urethral function; however, in most patients the initial operative procedure consists of biopsy done endoscopically, perineally, suprapubically, or occasionally by laparotomy. Once the diagnosis is confirmed, pretreatment staging of tumor size by CT scanning and cystogram are required. If laparotomy is performed, iliac and para-aortic node sampling should be included, as well as biopsies of any other clinically involved nodes. After diagnosis and pretreatment staging, chemotherapy and radiotherapy are initiated. Neoadjuvant therapy frequently results in tumor shrinkage and necrosis.[66,67,36] After several months of medical therapy, extent of disease should be reevaluated. If complete resection is possible surgeons should proceed to operation. Partial cystectomy has resulted in similar survival rates and higher rates of functional bladders compared with other treatment options.[36,67] Partial cystectomy usually is performed for bladder-dome tumors but may be applicable to more distal bladder lesions. These distal bladder resections may require ureteral reimplantation or bladder augmentation. Rarely, the response of prostatic tumors to neoadjuvant therapy may allow prostatectomy to be performed with complete tumor removal and preservation of the urethra and bladder. Prostate RMS more commonly requires prostatectomy, bladder salvage, and ureteral reconstruction.[68] Complete response to nonoperative therapy may not be rapid, so as long as there is continued partial response, radical resection (e.g., pelvic exenteration) should be delayed. If the tumor is still unresectable or not responding to medical therapy, then radical resection of all disease should be done and the patient should be provided with a continent urinary diversion. The treatment algorithm of biopsy, medical therapy, then second-look operation has improved survival (60–80%) and bladder preservation rates (83%).[36,67,70,71,26] RMS arising from the bladder has a better prognosis than prostrate probably because of ease and completeness of tumor excision.

*Paratesticular*

Paratesticular RMS usually presents early as Group I disease that is resected easily.[72,73] Most tumors present as painless scrotal masses.[74] Paratesticular RMS tumors are usually a variant of embryonal histology called spindle cell that has a very good prognosis with survival rates >90% for Groups I and II patients.[75,76] Lesions adjacent to the testis or spermatic cord should be removed by orchidectomy and resection of the entire spermatic cord through an inguinal incision with proximal control of the spermatic cord. The contralateral testis should be transposed to the adjacent thigh, temporarily, when scrotal radiotherapy is required. Open biopsy or tumor spillage of any kind should be avoided because inguinal recurrence may follow. If biopsy is believed necessary before orchidectomy, the following steps should be followed: (1) atraumatic high control of the spermatic cord; (2) mobilize the testis and cord carefully isolated from the operative field using a nonpermeable plastic bag; (3) biopsy site closed and testes covered while awaiting frozen section report; (4) instruments used for biopsy, gowns, and gloves changed; (5) if biopsy report is positive, testes and the entire cord including the atraumatic clamp should be immediately removed without removing the protective dressing or atraumatic clamp; (6) the field should be thoroughly irrigated. Patients with unprotected spillage are considered Clinical Group IIa regardless of the completeness of resection.

The incidence of nodal metastatic disease for paratesticular RMS is 26 - 43%.[77,73,78] All patients with paratesticular primary tumors should have thin-cut abdominal and pelvic CT scans with IV and PO contrast to evaluate for evidence of nodal involvement. Regional lymph nodes are the ipsilateral iliac and retroperitoneal nodes up to the upper pole of the ipsilateral kidney. Any suspicious nodes on CT scan should be considered positive (Group IIb) unless pathologically proven to be negative. However, studies have found that retroperitoneal lymph node status staged with CT scans may be incorrect for 58% of patients.[79] A review of IRSG patients indicated that lymph node sampling is not necessary in children less than 10 years old who have paratesticular RMS and negative CT scans. However, patients with enlarged nodes on CT scans, or children older than 10 years are required to have careful systematic ipsilateral nerve-sparing retroperitoneal node dissection with pathological evaluation for metastatic disease. These two groups of patients, enlarged nodes or children older than 10, have a much higher incidence of node positivity compared with other paratesticular patients (Weiner, in preparation). Suprarenal nodes should be incorporated into this sample because positive nodes higher than the renal vessels are considered disseminated metastatic disease and patients are classified as Group IV. Inguinal nodes rarely are involved and are biopsied only if clinically positive or if the scrotum is invaded by tumor. Inguinal nodes are not considered regional, and having positive nodes changes the patient to Group IV.

Resection of the hemiscrotal skin and contents are required when there is tumor fixation or invasion, or when a prior trans-scrotal biopsy has been performed. When the tumor has been inappropriately biopsied or removed by a trans-scrotal approach, a second operation is required including excision of the hemiscrotum and spermatic cord structures to the internal ring by an inguinal approach.

*Genitourinary: vulva, vagina, and uterus*

Usually these tumors are botryoid variants of embryonal RMS and have good prognoses, with survival >90%.[80] In the past, initial excision of a vaginal tumor often required radical procedures because of their large size at presentation. However, vulva/vagina/uterus RMS responds well to chemotherapy, with impressive tumor regression that generally precludes the need for radical operations such as pelvic exenteration. Therefore, these patients should be treated with initial biopsies followed by aggressive chemotherapy and radiotherapy with reevaluation for residual disease after several cycles of chemotherapy. Only 13–30% of patients treated with this plan required subsequent excision of a residual mass.[63,34] None had viable tumor in the resected specimens. Given these excellent results, the goal of surgery is local tumor resection while maintaining function.[81,82] Usually

**Table 4.** *Risk assignment according to group, stage and histology: IRS-V*

| Risk | Stage | Group | Site | Size | Age | Histology [1] | Metastasis | Nodes |
|---|---|---|---|---|---|---|---|---|
| Low A | | | | | | | | |
| D9602 | 1 | I | Favorable | a or b | <21 | EMB | M0 | N0 |
| | 1 | II | Favorable | a or b | <21 | EMB | M0 | N0 |
| | 1 | III | Orbit only | a or b | <21 | EMB | M0 | N0 |
| | 2 | I | Unfavorable | a | <21 | EMB | M0 | N0 or NX |
| Low B | | | | | | | | |
| D9602 | 1 | II | Favorable | a or b | <21 | EMB | M0 | N1 |
| | 1 | III | Orbit only | a or b | <21 | EMB | M0 | N1 |
| | 1 | III | Favorable (excluding orbit) | a or b | <21 | EMB | M0 | N0 or N1 or NX |
| | 2 | II | Unfavorable | a | <21 | EMB | M0 | N0 or NX |
| | 3 | I or II | Unfavorable | a | <21 | EMB | M0 | N1 |
| | 3 | I or II | Unfavorable | b | <21 | EMB | M0 | N0 or N1 or NX |
| Intermediate | | | | | | | | |
| (D9803) | 2 | III | Unfavorable | a | <21 | EMB | M0 | N0 or N*x* |
| | 3 | III | Unfavorable | a | <21 | EMB | M0 | N1 |
| | 3 | III | Unfavorable | a | <21 | EMB | M0 | N0 or N1 or N*x* |
| | 1 or 2 or 3 | I or II or III | Favorable or unfavorable | a or b | <21 | ALV | M0 | N0 or N1 or N*x* |
| | 4 | I or II or III or IV | Favorable or unfavorable | a or b | <10 | EMB | M1 | N0 or N1 |
| High | | | | | | | | |
| (D9802) | 4 | IV | Favorable or unfavorable | a or b | ≥10 | EMB | M1 | N0 or N1 |
| | 4 | IV | Favorable or unfavorable | a or b | <21 | ALV | M1 | N0 or N1 |

*Definitions:* Favorable = orbit/eyelid, head and neck (excluding parameningeal), genitourinary (not bladder or prostate). Unfavorable = bladder, prostate, extremity, parameningeal, other (trunk, retroperitoneal, etc.)
a = tumor size < = 5 cm in diameter; b = tumor size >5 cm in diameter.
EMB = embryonal, botryoid, or spindle variants or ectomesenchymomas with embryonal features. ALV = alveolar or undifferentiated sarcomas, or ectomesenchymomas with alveolar features.
N0 = regional nodes not clinically involved; N1 = regional nodes clinically involved; NX = node status unknown.

vaginectomy and hysterectomy only need to be performed for persistent or recurrent disease. Primary uterine tumors might be less responsive to chemotherapy than vaginal tumors and more often require aggressive resection. For uterine lesions treated with hysterectomies, preservation of the distal vagina and ovaries is usually possible. Direct tumor involvement is the only indication for oophorectomy.

### Metastatic disease

RMS most commonly metastasizes to lungs, bones, brain, liver, and lymph nodes by hematogenous and lymphatic routes. Unlike osteogenic sarcomas and some other soft tissue sarcomas, RMS and Ewing sarcoma are relatively sensitive to chemotherapy and radiotherapy. Therefore, although resection of metastatic disease for osteogenic sarcoma has been beneficial, the same advantage has not been shown for RMS.[83,84] The only indication for resection of metastatic disease is for pathological diagnosis or removal of localized, unresponsive tumor.

### Prognosis

Many factors influence long-term survival of patients with RMS, including stage, clinical group, disease site, tumor size, patient age, tumor histology, and distant metastatic disease. So many disparate prognostic factors have been elucidated that a risk classification system has been devised that encompasses all those variables (Table 4). The importance of accurate risk classification and indicated therapy cannot be over emphasized because appropriate initial therapy is a patient's best chance for cure. The salvage rate after relapse is a dismal 10 - 15%.[85] To facilitate correct placement of patients in IRSG protocols a web site has been developed that will determine the best protocol for each patient. Data from the patient is entered and the appropriate protocol is determined. The web site for this program is www.geocities. com/weisburd_marina/Home.html.

### References

1 Young JL Jr, Miller RW. Incidence of malignant tumors in U.S. children. *J Pediatr* 1975; 86: 254–8.
2 Ruymann FB. Rhabdomyosarcoma in children and adolescents: a review. *Hematol Oncol Clin North Am* 1987; 1: 621–54.
3 Pack G, Eberhart W. Rhabdomyosarcoma of the skeletal muscle: report of 100 cases. *Surgery* 1952; 32: 1023.
4 Green DM, Jaffe N. Progress and controversy in the treatment of childhood rhabdomyosarcoma. *Cancer Treat Rev* 1978; 5: 7–27.
5 Pinkel D, Pinkren J. Rhabdomyosarcoma in children. *J Am Med Assoc* 1961; 175: 293.
6 Edland R. Embryonal rhabdomyosarcoma. *Am J Roentgenol Radium Ther Nucl Med* 1965; 93: 671.
7 Crist W, Gehan EA, Ragab AH, *et al.* The Third Intergroup Rhabdomyosarcoma Study. *J Clin Oncol* 1995; 13: 610–30.
8 Maurer HM, Donaldson SS, Wiener ES. Rhabdomyosarcoma. In: Holland JF, Bast RC Jr, Morton DL, Frei E III, Kufe DW, Wechselbaum RR, eds. *Cancer Medicine.* 4th Edn. Vol 2. Baltimore, MD: William and Wilkins, 1997: Chapter 169.
9 Li FP, Fraumeni JF Jr, Mulvihill JJ, *et al.* A cancer family syndrome in twenty-four kindreds. *Cancer Res* 1988; 48: 5358–62.
10 Eng C, Schneider K, Fraumeni JF Jr, Li FP. Third international workshop on collaborative interdisciplinary studies of p53 and other predisposing genes in Li-Fraumeni syndrome. *Cancer Epidemiol Biomarkers Prev* 1997; 6: 379–83.
11 Grufferman A, *et al.* In utero X-ray exposure and risk of childhood rhabdomyosarcoma, Society for Epidemiologic Research 4th Annual Meeting, Buffalo, NY, 1991.
12 Grufferman S, Schwartz AG, Ruymann FB, Maurer HM. Parents' use of cocaine and marijuana and increased risk of rhabdomyosarcoma in their children. *Cancer Causes Control* 1993; 4: 217–24.
13 McKeen EA, Bodurtha J, Meadows AT, Douglass EC, Mulvihill JJ. Rhabdomyosarcoma complicating multiple neurofibromatosis. *J Pediatr* 1978; 93: 992–3.
14 Becker H, Zaunschirm A, Muntean W, Domej W. AlkohoLembryopathie und maligner tumor. *Wien Klin Wochenschr* 1982; 94: 364–5.
15 Newton WA, Hamoudi A, Webber B, Dickman PS. Pathology of Rhabdomyosarcoma and related tumors: experience of the Intergroup Rhabdomyosarcoma Studies. In: Maurer HM, Ruymann FB, Pochedly C, eds. *Rhabdomyosarcoma and related tumors in children and adolescents*. Boca Raton, FL: CRC Press, 1991: 19–48.
16 McHugh K, Boothroyd AE. The role of radiology in childhood rhabdomyosarcoma. *Clin Radiol* 1999; 54: 2–10.
17 Mafee MF, Pai E, Philip B. Rhabdomyosarcoma of the orbit. Evaluation with MR imaging and CT. *Radiol Clin North Am* 1998; 36: 1215–27, xii.
18 Sordillo PP, Reiman RE, Gelbard AS, Benua RS, Magill GB, Laughlin JS. Scanning with L-(13 N) glutamate: Assessment of the response to chemotherapy of a patient with embryonal rhabdomyosarcoma. *Am J Clin Oncol* 1982; 5: 285–9.
19 Lawrence W Jr, Gehan EA, Hays DM, Beltangady M, Maurer HM. Prognostic significance of staging factors of the UICC staging system in childhood rhabdomyosarcoma: a report from the Intergroup Rhabdomyosarcoma Study (IRS-II). *J Clin Oncol* 1987; 5: 46–54.
20 Lawrence W Jr, Anderson JR, Gehan EA, Maurer H. Pretreatment TNM staging of childhood rhabdomyosarcoma: a report of the Intergroup Rhabdomyosarcoma Study Group. Children's Cancer Study Group. Pediatric Oncology Group. *Cancer* 1997; 80: 1165–70.
21 Salomao DR, Sigman JD, Greenebaum E, Cohen MB. Rhabdomyosarcoma presenting as a parotid gland mass in pediatric patients: fine-needle aspiration biopsy findings. *Cancer* 1998; 84: 245–51.
22 Layfield LJ. Fine-needle aspiration of the head and neck. *Pathology (Phila)* 1996; 4: 409–38.
23 Hays DM. New approaches to the surgical management of rhabdomyosarcoma in childhood. *Chir Pediatr* 1990; 31: 197–201.
24 Wiener ES. Rhabdomyosarcoma: new dimensions in management. *Semin Pediatr Surg* 1993; 2: 47–58.
25 Atahan S, Aksu O, Ekinci C. Cytologic diagnosis and subtyping of rhabdomyosarcoma. *Cytopathology* 1998; 9: 389–97.

26 Lobe TE, Wiener E, Andrassy RJ, *et al.* The argument for conservative, delayed surgery in the management of prostatic rhabdomyosarcoma. *J Pediatr Surg* 1996; 31: 1084–7.

27 Neville HL, Andrassy RJ, Lobe TE, *et al.* Preoperative staging, prognostic factors, and outcome for extremity rhabdomyosarcoma: a preliminary report from the Intergroup Rhabdomyosarcoma Study IV (1991–1997). *J Pediatr Surg* 2000; 35: 317–21.

28 Lawrence W Jr, Hays DM, Heyn R, *et al.* Lymphatic metastases with childhood rhabdomyosarcoma. A report from the Intergroup Rhabdomyosarcoma Study. *Cancer* 1987; 60: 910–5.

29 Neville HL, Andrassy RJ, Lally KP, Corpron C, Ross MI. Lymphatic mapping with sentinel node biopsy in pediatric patients. *J Pediatr Surg* 2000; 35: 961–4.

30 Hays DM, Lawrence W Jr, Wharam M, *et al.* Primary reexcision for patients with 'microscopic residual' tumor following initial excision of sarcomas of trunk and extremity sites. *J Pediatr Surg* 1989; 24: 5–10.

31 Hays DM, Raney RB, Crist WM, *et al.* Secondary surgical procedures to evaluate primary tumor status in patients with chemotherapy-responsive stage III and IV sarcomas: a report from the Intergroup Rhabdomyosarcoma Study. *J Pediatr Surg* 1990; 25: 1100–5.

32 Baker KS, Anderson JR, Link MP, *et al.* Benefit of intensified therapy for patients with local or regional embryonal rhabdomyosarcoma: results from the Intergroup Rhabdomyosarcoma Study IV. *J Clin Oncol* 2000; 18: 2427–34.

33 Shields JA, Shields CL. Rhabdomyosarcoma of the orbit. *Int Ophthalmol Clin* 1993; 33: 203–10.

34 Andrassy RJ, Wiener ES, Raney RB, *et al.* Progress in the surgical management of vaginal rhabdomyosarcoma: a 25-year review from the Intergroup Rhabdo-myosarcoma Study Group. *J Pediatr Surg* 1999; 34: 731–5.

35 Spunt SL, Lobe TE, Pappo AS, *et al.* Aggressive surgery is unwarranted for biliary tract rhabdomyosarcoma. *J Pediatr Surg* 2000; 35: 309–16.

36 Heyn R, Newton WA, Raney RB, *et al.* Preservation of the bladder in patients with rhabdomyosarcoma. *J Clin Oncol* 1997; 15: 69–75.

37 Tabrizi P, Letts M. Childhood rhabdomyosarcoma of the trunk and extremities. *Am J Orthop* 1999; 28: 440–6.

38 Crist WM, Garnsey L, Beltangady MS, *et al.* Prognosis in children with rhabdomyosarcoma: a report of the intergroup rhabdomyosarcoma studies I and II. Intergroup Rhabdomyosarcoma Committee. *J Clin Oncol* 1990; 8: 443–52.

39 Wiener E. Second look operations in children in group III and IV rhabdomyosarcoma. *Med Pediatr Oncol* 1990; 18: 408.

40 Wiener ES, Hays DM. Rhabdomyosarcoma in extremity and trunk sites. In: Maurer HM, Ruymann FB, Pochedly C, eds. *Rhabdomyosarcoma and related tumors in children and adolescents*. Boca Raton, FL: CRC Press, 1991: 363–72.

41 Wiener E, Lawrence W, Hays D, Fryer C, Ortega J, Newton W, Johnston J, Gehan E, Maurer H. Complete response or not complete response? Second look operations are the answer in children with rhabdomyosarcoma. *Proc Am Soc Clin Oncol* 1991; 10: 316.

42 Kinsella TJ, Miser JS, Triche TJ, Horvath K, Glatstein E. Treatment of high-risk sarcomas in children and young adults: analysis of local control using intensive combined modality therapy. *Natl Cancer Inst Monogr* 1988; 6: 291–6.

43 Tefft M, Lindberg RD, Gehan EA. Radiation therapy combined with systemic chemotherapy of rhabdomyosarcoma in children: local control in patients enrolled in the Intergroup Rhabdomyosarcoma Study. *Natl Cancer Inst Monogr* 1981; 75–81.

44 Wiener ES. Head and neck rhabdomyosarcoma. *Semin Pediatr Surg* 1994; 3: 203–6.

45 Anderson GJ, Tom LW, Womer RB, Handler SD, Wetmore RF, Potsic WP. Rhabdomyosarcoma of the head and neck in children. *Arch Otolaryngol Head Neck Surg* 1990; 116: 428–31.

46 Gross M, Gutjahr P. Therapy of rhabdomyosarcoma of the larynx. *Int J Pediatr Otorhinolaryngol* 1988; 15: 93–7.

47 Daya H, Chan HS, Sirkin W, Forte V. Pediatric rhabdomyosarcoma of the head and neck: is there a place for surgical management? *Arch Otolaryngol Head Neck Surg* 2000; 126: 468–72.

48 Blatt J, Snyderman C, Wollman MR, *et al.* Delayed resection in the management of non-orbital rhabdomyosarcoma of the head and neck in childhood. *Med Pediatr Oncol* 1997; 28: 294–8.

49 Lyos AT, Goepfert H, Luna MA, Jaffe N, Malpica A. Soft tissue sarcoma of the head and neck in children and adolescents. *Cancer* 1996; 77: 193–200.

50 Raney RB, Anderson JR, Kollath J, *et al.* Late effects of therapy in 94 patients with localized rhabdomyosarcoma of the orbit: Report from the Intergroup Rhabdomyosarcoma Study (IRS)-III, 1984–1991. *Med Pediatr Oncol* 2000; 34: 413–20.

51 Alvarez Silvan AM, Garcia Canton JA, Pineda Cuevas G, Alfuro Gutierrez J. Successful treatment of orbital rhabdomyosarcoma in two infants using chemotherapy alone. *Med Pediatr Oncol* 1996; 26: 186–9.

52 Notis CM, Abramson DH, Sagerman RH, Ellsworth RM. Orbital rhabdomyosarcoma: treatment or overtreatment. *Ophthalm Genet* 1995; 16: 159–62.

53 Raney RB Jr, Ragab AH, Ruymann FB, *et al.* Soft-tissue sarcoma of the trunk in childhood. Results of the intergroup rhabdomyosarcoma study. *Cancer* 1982; 49: 2612–6.

54 Ortega JA, Wharam M, Gehan EA, *et al.* Clinical features and results of therapy for children with paraspinal soft tissue sarcoma: a report of the Intergroup Rhabdomyosarcoma Study. *J Clin Oncol* 1991; 9: 796–801.

55 Sundaresan N, Rosen G, Fortner JG, Lane JM, Hilaris BS. Preoperative chemotherapy and surgical resection in the management of posterior paraspinal tumors. Report of three cases. *J Neurosurg* 1983; 58: 446–50.

56 Beech TR, Moss RL, Anderson JA, *et al.* What comprises appropriate therapy for children/adolescents with rhabdomyosarcoma arising in the abdominal wall? A report from the Intergroup Rhabdomyosarcoma Study Group. *J Pediatr Surg* 1999; 34: 668–71.

57 Andrassy RJ, Wiener ES, Raney RB, *et al.* Thoracic sarcomas in children. *Ann Surg* 1998; 227: 170–3.

58 Ito F, Watanabe Y, Harada T, *et al.* Combined resection of abdominal aorta and inferior vena cava for retroperitoneal rhabdomyosarcoma invading the aortoiliac bifurcation. *J Pediatr Surg* 1998; 33: 1566–8.

59 Blakely ML, Lobe TE, Anderson JR, *et al.* Does debulking improve survival rate in advanced-stage retroperitoneal embryonal rhabdomyosarcoma? *J Pediatr Surg* 1999; 34: 736–42.

60 Pollono DG, Tomarchio S, Berghoff R, Drut R, Urrutia A, Cedola J. Rhabdomyosarcoma of extrahepatic biliary tree: initial treatment with chemotherapy and conservative surgery. *Med Pediatr Oncol* 1998; 30: 290–3.

61 Sanz N, de Mingo L, Florez F, Rollan V. Rhabdomyosarcoma of the biliary tree. *Pediatr Surg Int* 1997; 12: 200–1.

62 Lawrence W Jr, Hays DM, Heyn R, Beltangady M, Maurer HM. Surgical lessons from the Intergroup Rhabdomyosarcoma Study (IRS) pertaining to extremity tumors. *World J Surg* 1988; 12: 676–84.

63 Andrassy RJ, Corpron CA, Hays D, *et al.* Extremity sarcomas: an analysis of prognostic factors from the Intergroup Rhabdomyosarcoma Study III. *J Pediatr Surg* 1996; 31: 191–6.
64 Breneman JC, Wiener ES. Issues in the local control of rhabdomyosarcoma. *Med Pediatr Oncol* 2000; 35: 104–9.
65 Mandell L, Ghavimi F, LaQuaglia M, Exelby P. Prognostic significance of regional lymph node involvement in childhood extremity rhabdomyosarcoma. *Med Pediatr Oncol* 1990; 18: 466–71.
66 Merguerian PA, Agarwal S, Greenberg M, Bagli DJ, Khoury AE, McLorie GA. Outcome analysis of rhabdomyosarcoma of the lower urinary tract. *J Urol* 1998; 160: 1191–4; discussion 1216.
67 Silvan AM, Gordillo MJ, Lopez AM, *et al.* Organ-preserving management of rhabdomyosarcoma of the prostate and bladder in children. *Med Pediatr Oncol* 1997; 29: 573–5.
68 Hays DM, Raney RB, Wharam MD, *et al.* Children with vesical rhabdomyosarcoma (RMS) treated by partial cystectomy with neoadjuvant or adjuvant chemotherapy, with or without radiotherapy. A report from the Intergroup Rhabdomyosarcoma Study (IRS) Committee [published erratum appears in *J Pediatr Hematol Oncol* 1995; 17: 356]. *J Pediatr Hematol Oncol* 1995; 17: 46–52.
69 Lander EB, Shanberg AM, Tansey LA, Sawyer DE, Groncy PK, Finklestein JZ. The use of continent diversion in the management of rhabdomyosarcoma of the prostate in childhood. *J Urol* 1992; 147: 1602–5.
70 La Quaglia MP, Ghavimi F, Herr H, *et al.* Prognostic factors in bladder and bladder-prostate rhabdomyosarcoma. *J Pediatr Surg* 1990; 25: 1066–72.
71 Raney B Jr, Heyn R, Hays DM, *et al.* Sequelae of treatment in 109 patients followed for 5 to 15 years after diagnosis of sarcoma of the bladder and prostate. A report from the Intergroup Rhabdomyosarcoma Study Committee. *Cancer* 1993; 71: 2387–94.
72. Raney RB Jr, Hays DM, Lawrence W Jr, Soule EH, Tefft M, Donaldson MH. Paratesticular rhabdomyosarcoma in childhood. *Cancer* 1978; 42: 729–36.
73 Raney RB Jr, Tefft M, Lawrence W Jr, *et al.* Paratesticular sarcoma in childhood and adolescence. A report from the Intergroup Rhabdomyosarcoma Studies I and II, 1973–1983. *Cancer* 1987; 60: 2337–43.
74 Sugita Y, Clarnette TD, Cooke-Yarborough C, Chow CW, Waters K, Hutson JM. Testicular and paratesticular tumours in children: 30 years' experience. *Aust N Z J Surg* 1999; 69: 505–8.
75 Ferrari A, Casanova M, Massimino M, Luksch R, Piva L, Fossati-Bellani F. The management of paratesticular rhabdomyosarcoma: a single institutional experience with 44 consecutive children. *J Urol* 1998; 159: 1031–4.
76 Leuschner I, Newton WA Jr, Schmidt D, *et al.* Spindle cell variants of embryonal rhabdomyosarcoma of the paratesticular region. A report of the Intergroup Rhabdomyosarcoma Study. *Am J Surg Pathol* 1993; 17: 221–30.
77 Olive D, Flamant F, Zucker JM, *et al.* Paraaortic lymphadenectomy is not necessary in the treatment of localized paratesticular rhabdomyosarcoma. *Cancer* 1984; 54: 1283–7.
78 Wiener ES, Lawrence W, Hays D, *et al.* Retroperitoneal node biopsy in paratesticular rhabdomyosarcoma. *J Pediatr Surg* 1994; 29: 171–8.
79 Hermans BP, Foster RS, Bihrle R, *et al.* Is retroperitoneal lymph node dissection necessary for adult paratesticular rhabdomyosarcoma? *J Urol* 1998; 160: 2074–7.
80 Martelli H, Oberlin O, Rey A, *et al.* Conservative treatment for girls with nonmetastatic rhabdomyosarcoma of the genital tract: a report from the Study Committee of the International Society of Pediatric Oncology. *J Clin Oncol* 1999; 17: 2117–22.
81 Corpron CA, Andrassy RJ, Hays DM, *et al.* Conservative management of uterine pediatric rhabdomyosarcoma: a report from the Intergroup Rhabdomyosarcoma Study III and IV pilot. *J Pediatr Surg* 1995; 30: 942–4.
82 Andrassy RJ, Hays DM, Raney RB, *et al.* Conservative surgical management of vaginal and vulvar pediatric rhabdomyosarcoma: a report from the Intergroup Rhabdomyosarcoma Study III. *J Pediatr Surg* 1995; 30: 1034–7.
83 Temeck BK, Wexler LH, Steinberg SM, McClure LL, Horowitz M, Pass HI. Metastasectomy for sarcomatous pediatric histologies: results and prognostic factors. *Ann Thorac Surg* 1995; 59: 1385–90.
84 LaQuaglia MP. The surgical management of metastases in pediatric cancer. *Semin Pediatr Surg* 1993; 2: 75–82.
85. Arndt CA, Crist WM. Common musculoskeletal tumors of childhood and adolescence. *New Engl J Med* 1999; 341: 342–52.
